# Odontogenic Cutaneous Fistula Following Wisdom Tooth Extraction: A Rare Case Report

**DOI:** 10.7759/cureus.86256

**Published:** 2025-06-18

**Authors:** Antoine Bruneau, Jean Lehner, Chloé Bertolus, Anthony Levy-Bohbot

**Affiliations:** 1 Department of Oral Surgery, Hôpital Pitié Salpêtrière, Paris, FRA; 2 Département de Pathologie de la Muqueuse Buccale, Service de Chirurgie Maxillo-Faciale et Stomatologie, Service de Chirurgie Maxillo-Faciale et Stomatologie, Hôpital Pitié Salpêtrière, Paris, FRA

**Keywords:** infection, mandible, mandibular wisdom tooth, odontogenic cutaneous fistula, odontogenic sinus tract, oral diseases, oral surgery

## Abstract

Odontogenic cutaneous fistula is a rare condition that is frequently misdiagnosed. This article presents a case of an odontogenic cutaneous fistula following mandibular wisdom tooth removal. An 18-year-old woman with no underlying medical conditions presented to the oral and maxillofacial surgery department with swelling of the left cheek and a facial skin fistula. A surgical fistulectomy was performed under general anesthesia, and follow-up revealed satisfactory mucosal and skin healing, with residual cutaneous retraction.

## Introduction

Odontogenic cutaneous fistula results from dental infections and is defined as a communication between the skin and the oral cavity. Although the prevalence of cutaneous fistulas of odontogenic origin is unknown, they remain underreported in the literature, with only about ten case reports published over the past ten years, and none linked to a surgical procedure. Due to its rarity, the condition is often misdiagnosed by dermatologists and general practitioners [1].

We report the case of an 18-year-old woman who presented to the Oral and Maxillofacial Surgery Department at the Pitié-Salpêtrière Hospital in Paris, France, with an odontogenic cutaneous fistula following the extraction of a mandibular wisdom tooth.

While such lesions are frequently first assessed by dermatologists, this case is notable for the early post-surgical development of a cutaneous fistula, which was correctly diagnosed and managed by the oral surgery team.

The aim of this article is to highlight the diagnosis and treatment of a cutaneous lesion of odontogenic origin in a surgical setting following wisdom tooth extraction.

## Case presentation

**Figure 1:**
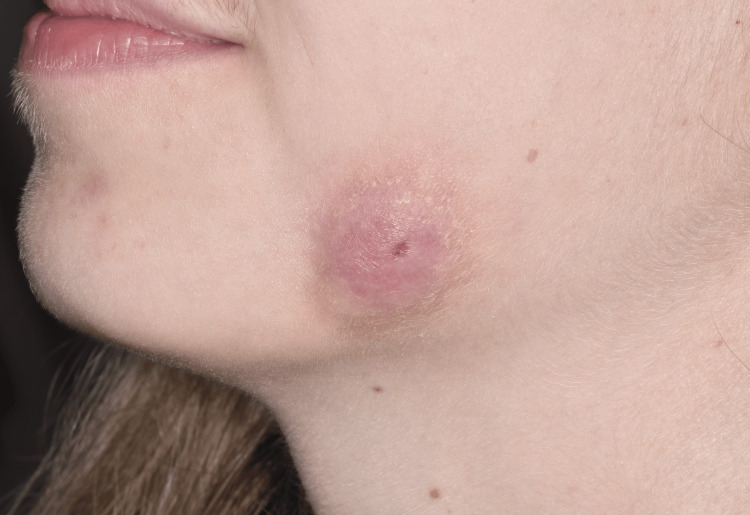
Photograph of the left cheek showing a 25 mm skin nodule.

An 18-year-old woman presented with a cutaneous lesion and swelling of the left cheek. Clinical examination revealed a 25 mm skin nodule with a central depression (Figure 1). The lesion was painless. The patient’s medical history was unremarkable, except for the removal of mandibular wisdom teeth three weeks prior to consultation. These teeth were extracted as a preventive measure, justified by episodes of pericoronitis experienced by the patient.

**Figure 2:**
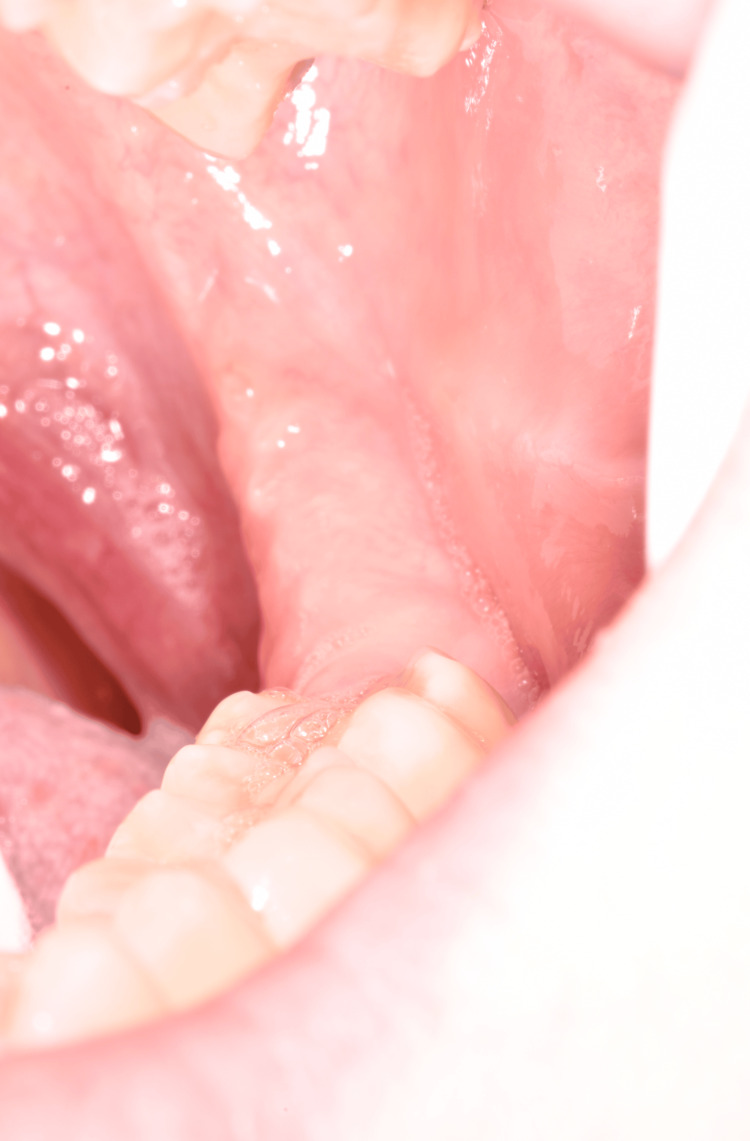
Intraoral photograph showing healed mucosa.

Intraoral examination showed normal mucosal healing at the extraction site (Figure 2). Following the clinical examination, several differential diagnoses were considered: a cutaneous fistula of odontogenic origin, a sebaceous cyst, or a carbuncle.

**Figure 3:**
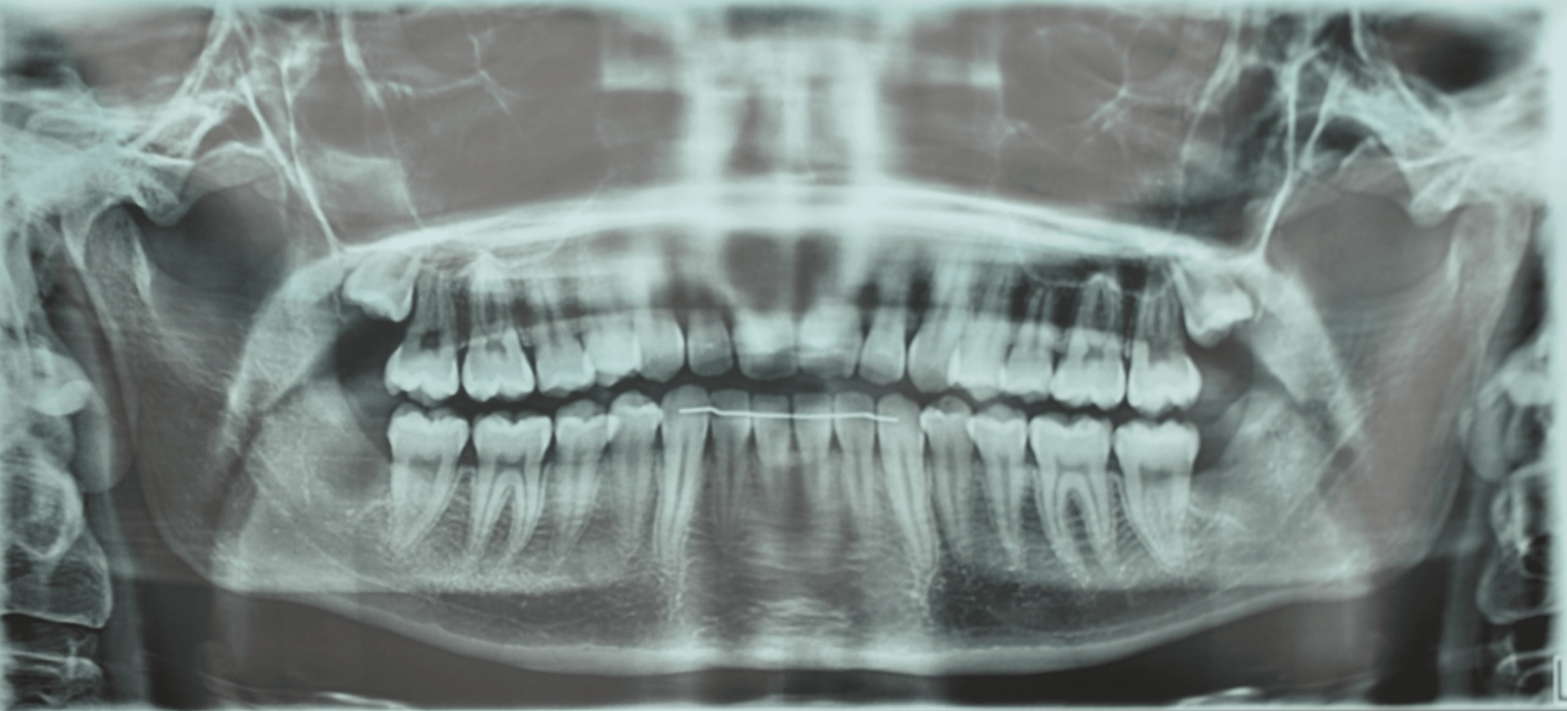
Panoramic X-ray.

**Figure 4:**
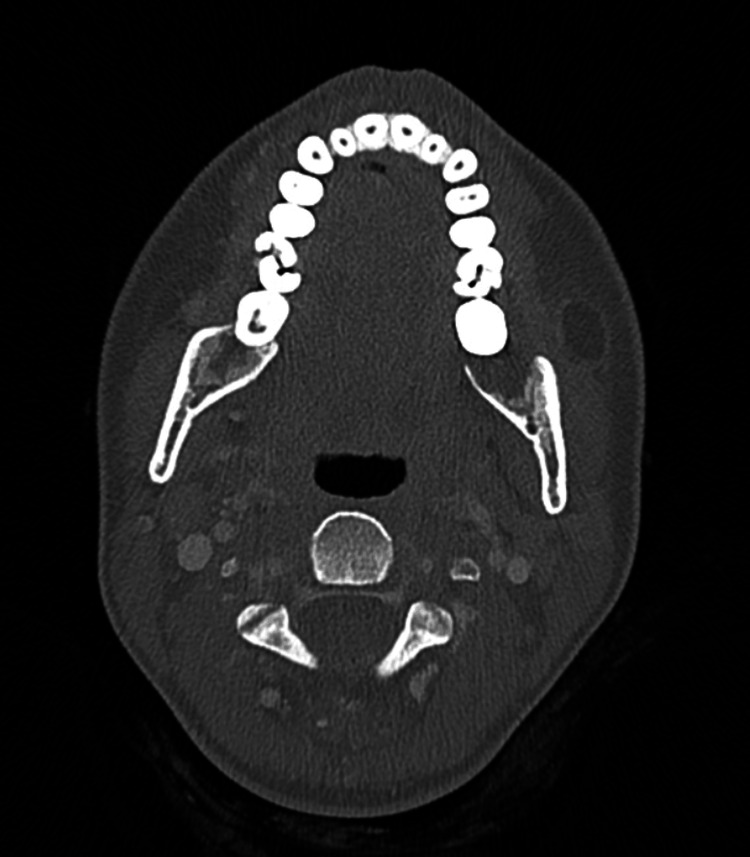
CT view.

Panoramic X-ray imaging was unremarkable (Figure 3), and CT of the head and neck revealed no abnormalities (Figure 4). 

**Figure 5:**
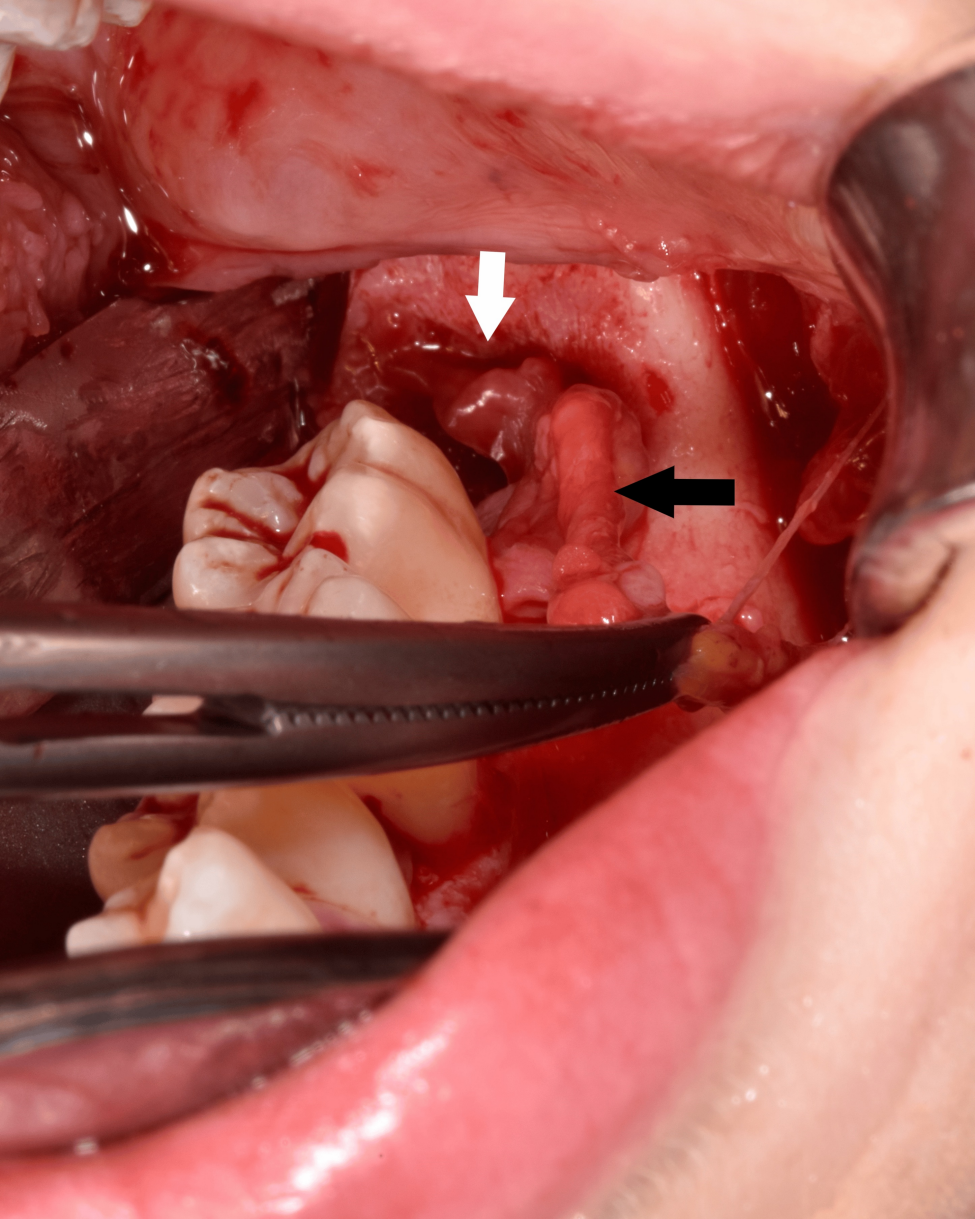
Intraoperative view. White arrow indicates the extraction socket; black arrow shows the fistula tract.

**Figure 6:**
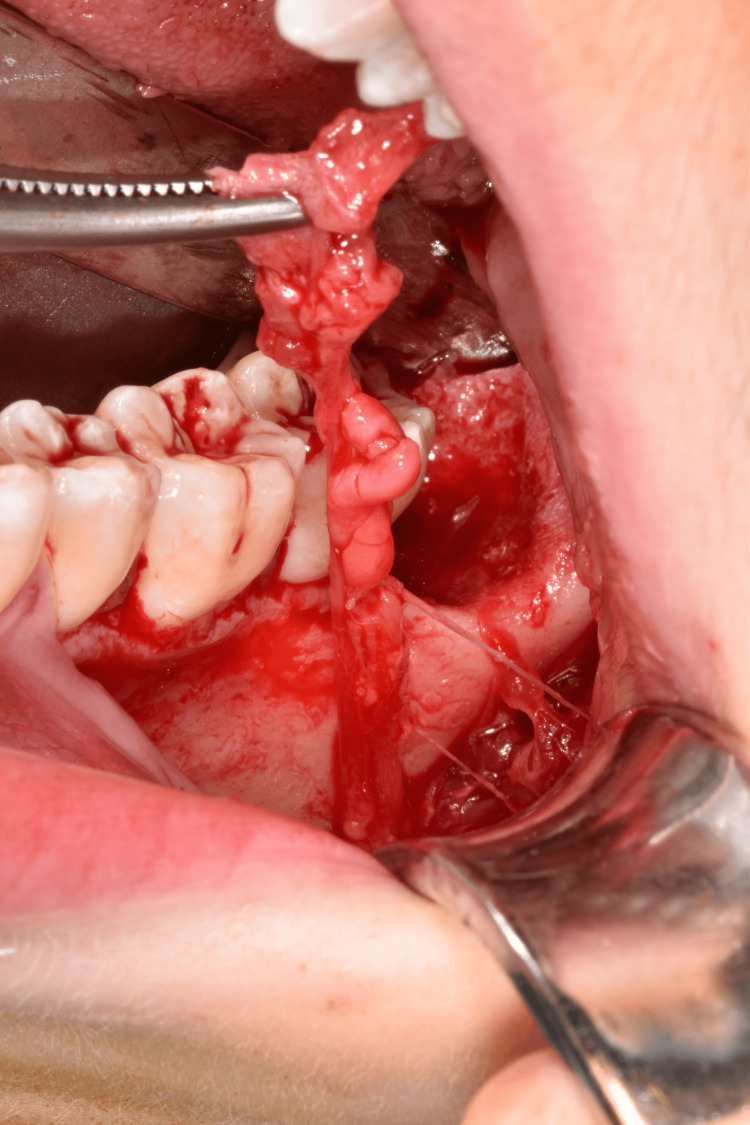
Dissection of the fistula tract.

**Figure 7:**
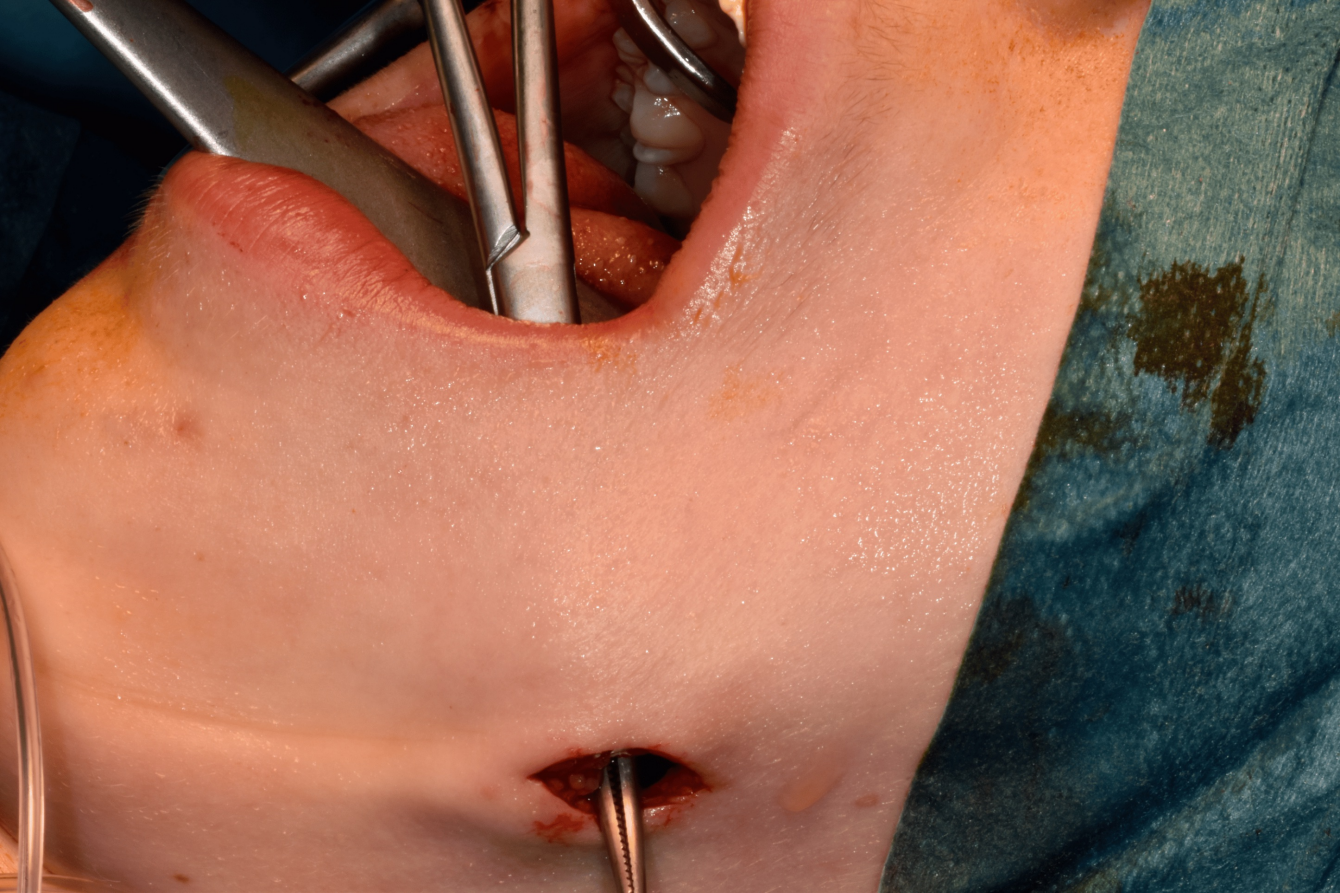
A Kelly forceps introduced intraorally showing the direction of the fistula.

The patient underwent fistulectomy under general anesthesia using a combined intraoral and extraoral approach. A sulcular incision was made from tooth 35 to 38, and a mucoperiosteal flap was raised to access the fistula (Figure 5). The fistula tract was dissected and removed along with granulation tissue through the buccinator muscle (Figures 6-7). The origin of the fistula from the post-extraction socket of the wisdom tooth confirmed its odontogenic nature. The surgical site was irrigated with povidone-iodine and saline solutions. Skin closure was achieved with non-absorbable sutures, and mucosal closure with absorbable sutures.

**Figure 8:**
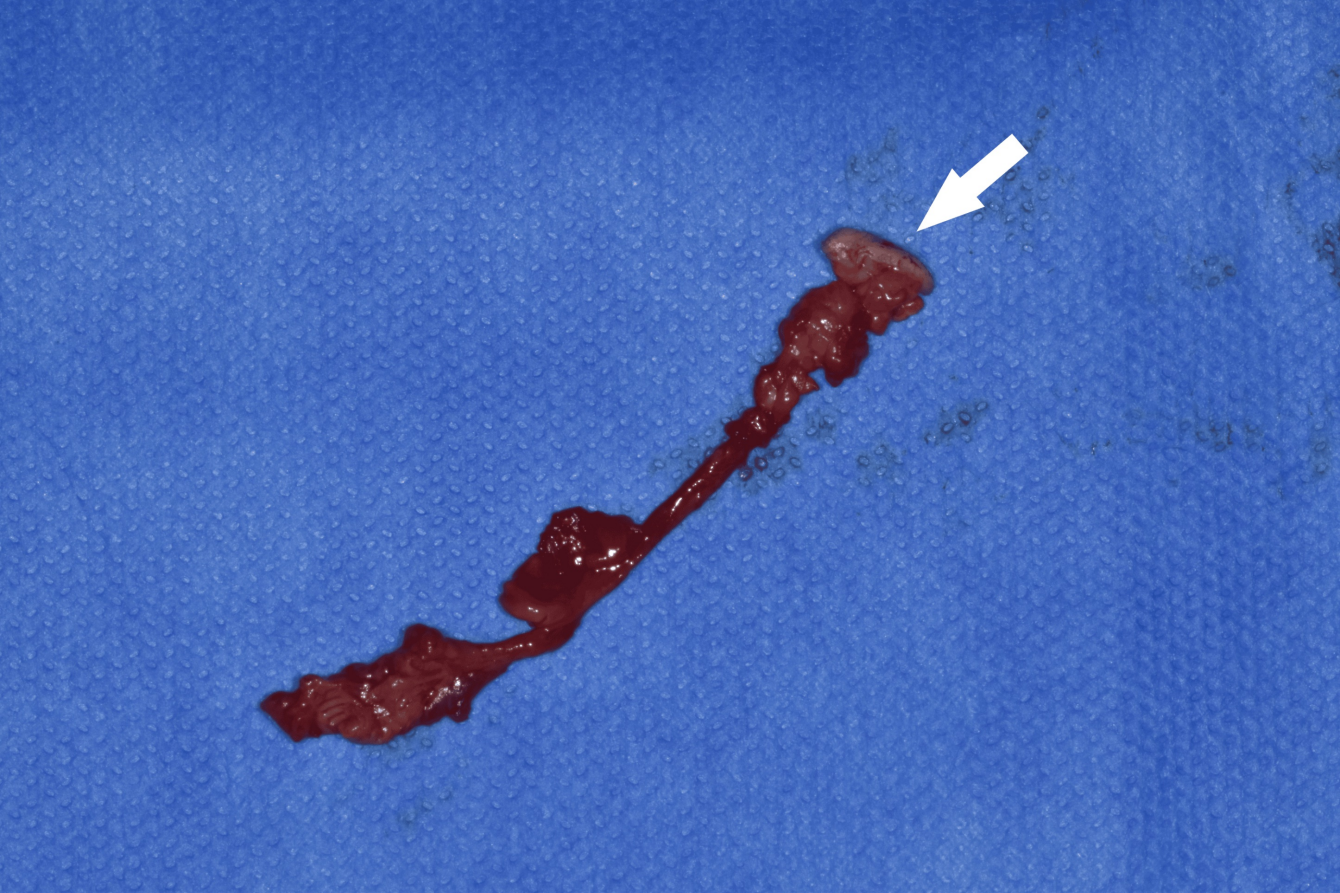
Operative resection specimen. The arrow indicates the skin segment resected.

**Figure 9:**
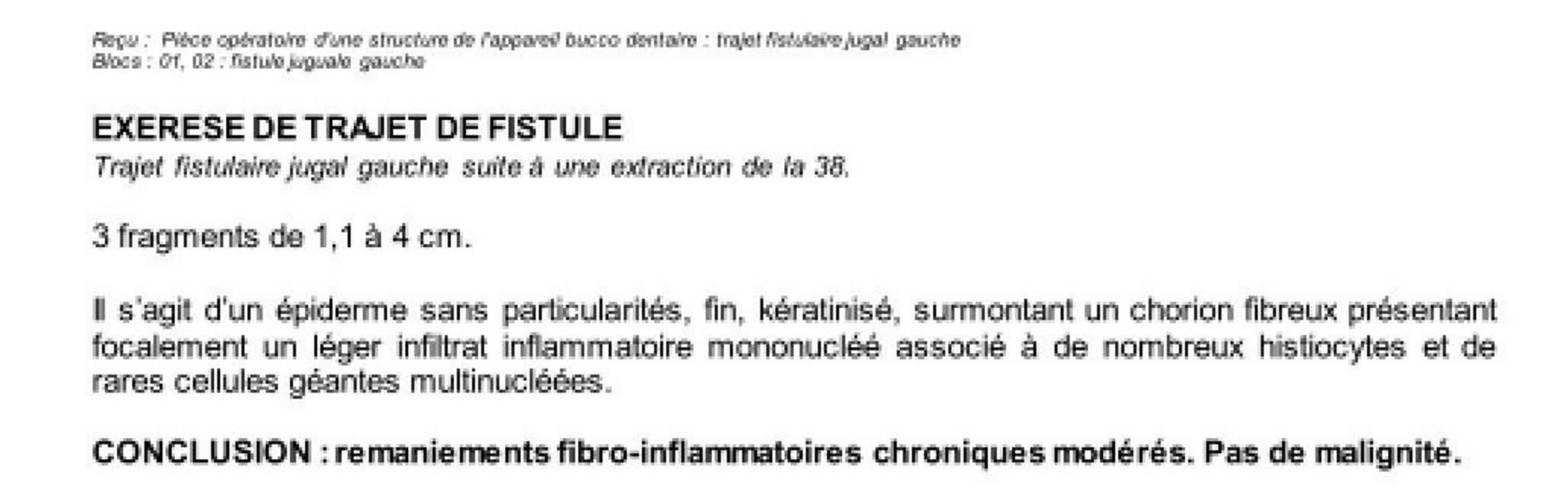
Histopathological report.

The excised fistula was sent for histopathological examination (Figures 8-9). Antibiotic therapy with amoxicillin and clavulanic acid was initiated after obtaining the antibiogram results from intraoperative bacteriological samples.

**Figure 10:**
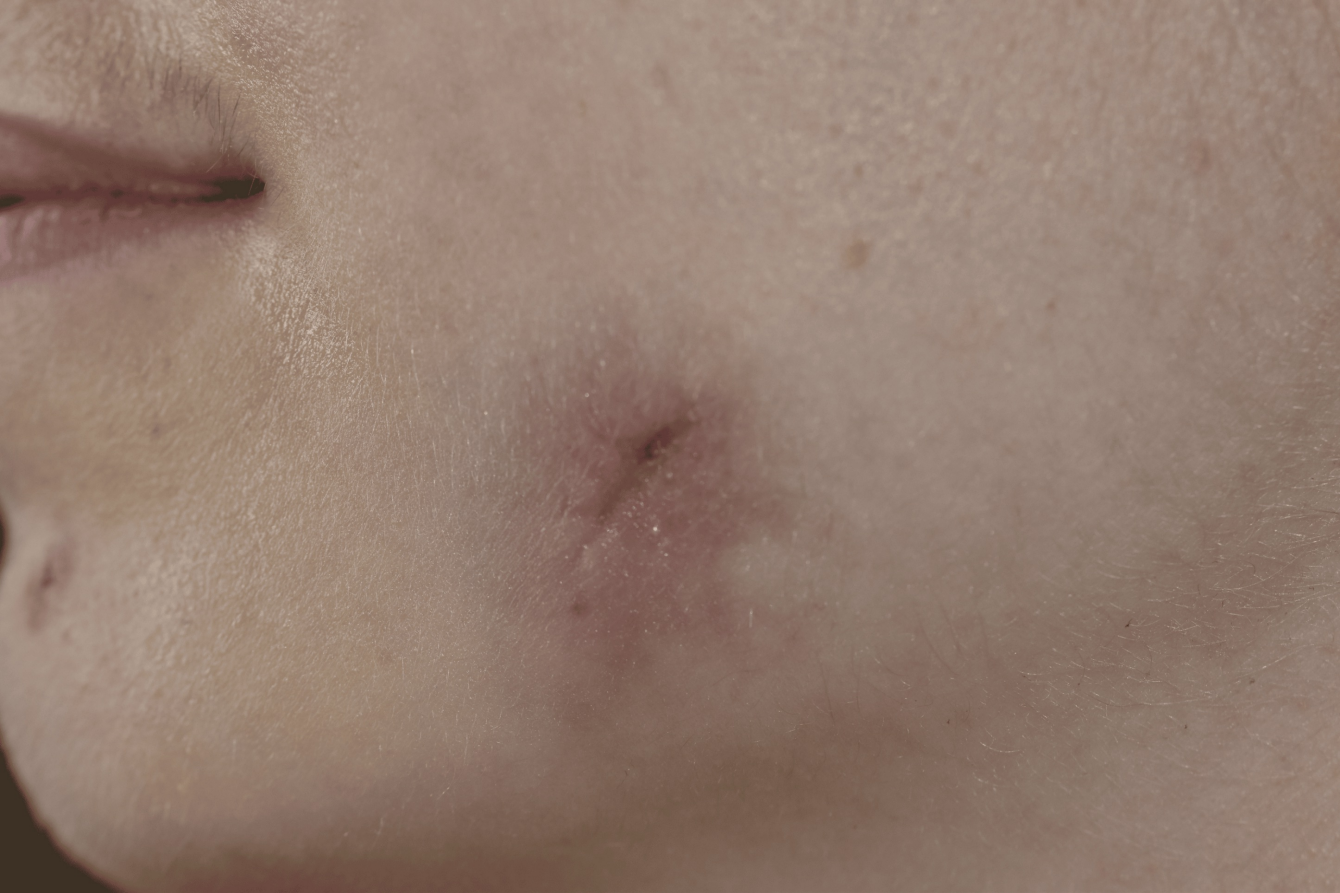
Skin healing at two weeks postoperatively.

Two weeks postoperatively, complete skin healing was observed, although mild residual cutaneous retraction remained (Figure 10). No postoperative swelling or infection occurred. Histopathological analysis revealed normal epidermis with fibrosis and an inflammatory cell reaction, with no evidence of malignancy.

## Discussion

Odontogenic cutaneous fistulas are rare complications of chronic dental infections [1]. These fistulas are characterized by a sinus tract that connects the skin to the alveolar bone via a musculo-mucosal pathway. The tract is composed of granulation tissue, and gentle pressure on the surrounding skin may elicit purulent discharge.

The odontogenic origin is supported here by the chronological proximity between the surgery and the appearance of the cutaneous fistula, as well as the fistulous tract originating within the socket of the extracted tooth. The low incidence of tuberculosis in France (approximately 4,000 cases per year), and particularly of osseous tuberculosis (100 to 200 cases per year), along with the low incidence of actinomycosis (100 to 200 cases per year), meant that these conditions were not initially considered as differential diagnoses.

Common causes include periodontal infections, retained root fragments after extraction, or pulpal degeneration [2]. They can also arise following bone-grafting procedures [3], peri-implantitis around dental implants [4], or root fractures [5].

The location of odontogenic cutaneous fistulas varies based on the affected tooth. Large case series, such as those by Guevara-Guevara-Gutiérrez E et al. [1] or Xia J et al. [6], report that between 64% and 76.7% of cases occur in the mandibular angle and chin region. Other studies have found similar results, with the mandible implicated in up to 80% of cases [7, 8].

Cutaneous fistulas of dental origin typically follow anatomical paths of least resistance, navigating between muscular structures and the periosteum. They emerge at the skin surface after traversing fascial and adipose spaces characterized by low mechanical resistance.

The clinical presentation and the course of the fistula, extending forward past the muscle to the premolar region, should suggest a rare form of migratory abscess known as Chompret-L’Hirondelle migratory abscess [9]. Typically, this type of migratory abscess occurs when a wisdom tooth or molar is still present. The fistulous tract usually forms extending forward beyond the buccinator muscle. In this case, however, the abscess and fistula developed after tooth extraction, with satisfactory healing of the buccal mucosa, which led to a delayed diagnosis and an unsuccessful attempt at medical treatment.

Clinical presentations range from dimpling nodules and abscesses to cysts, ulcers, or draining lesions, as described by Lee EY et al. [10]. In this case, the lesion appeared as a nodule. This variability can lead to misdiagnosis, as the lesion may mimic skin conditions such as epidermal cysts, furuncles, abscesses, or actinomycosis [11]. In the article by Lee [10], 81.8% of patients were initially misdiagnosed. Patients often first consult dermatologists or general practitioners, who may not suspect a dental origin.

Diagnostic evaluation should include a thorough intraoral examination and panoramic X-rays [12]. Fistulography with a gutta-percha cone can also aid in diagnosis [13]. Cone beam computed tomography (CBCT) may occasionally identify the fistula tract [14], though it was unremarkable in this case.

Surgical management involves complete fistulectomy and irrigation of the tract [15]. The underlying infection must be addressed through tooth extraction or endodontic treatment. Skin and mucosal closure should be achieved, supported by antibiotic therapy, antiseptic mouthwash, and careful postoperative care. The prognosis of oral cutaneous fistulas is excellent when treatment is initiated promptly [16].

## Conclusions

Odontogenic cutaneous fistulas are rare and pose a significant diagnostic challenge. This case highlights the importance of considering odontogenic etiologies in the differential diagnosis of atypical cutaneous facial lesions, particularly in the post-extraction context. A combined intraoral and extraoral surgical approach enabled complete excision of the fistulous tract and confirmed its odontogenic origin. Accurate differential diagnosis is essential to exclude other dermatologic or infectious conditions with similar clinical presentations. Histopathological analysis and culture-guided antibiotic therapy are crucial for ensuring resolution and preventing recurrence. Effective management requires addressing the underlying cause and performing surgical excision of the fistula.
